# Hemophagocytic Lymphohistiocytosis in a Remote Kidney Transplant Recipient Triggered by HSV Infection With Complete Recovery: An Educational Case Report

**DOI:** 10.1177/20543581241253921

**Published:** 2024-05-23

**Authors:** Anjana Gopal, S. Joseph Kim

**Affiliations:** 1 Ajmera Transplant Centre, Toronto General Hospital, University Health Network, Toronto, ON, Canada

**Keywords:** hemophagocytic lymphohistiocytosis, kidney transplant, herpes simplex virus

## Abstract

**Rationale:**

Hemophagocytic lymphohistiocytosis (HLH) is a life-threatening disease characterized by excessive immune activation. It is more commonly seen in children but increasingly recognized in adults. Primary HLH relies on a genetic predisposition, whereas secondary HLH develops in the context of infections, malignancies, or autoimmune diseases. Hemophagocytic lymphohistiocytosis has been rarely described in patients on immunosuppressive therapy after kidney transplant. Here, we describe a case of HLH in a patient with a remote history of kidney transplant, triggered by a viral infection.

**Presenting Concerns:**

A 45-year-old female, with a kidney transplant in 2009 for IgA nephropathy, presented with fever, vomiting, and back pain of 1-week duration. She was on triple immunosuppression consisting of daily doses of prednisone 5 mg, azathioprine 100 mg, and tacrolimus extended release 1 mg, and a baseline creatinine of 130 µmol/L.

**Diagnosis:**

Initial investigations showed anemia, leukopenia, elevated serum creatinine, transaminitis, and markedly increased ferritin of 67 600 µg/L which prompted a bone marrow biopsy to rule out HLH. The bone marrow showed an increased proportion of CD68+ cells (macrophages) with more than 5 in 1000 hemophagocytic macrophages. Her soluble IL-2 receptor (CD25) level was 3406 pg/mL (606-2299 pg/mL) which was mildly elevated. She fulfilled 4 of the 8 criteria for HLH and with an H score was 223 which suggested a diagnosis of HLH with 96.9% probability. An extensive secondary workup for possible triggers for HLH led to a swab from genital ulcers that was positive for herpes simplex virus (HSV) type 2. The polymerase chain reaction (PCR) in the blood for HSV type 2 was also positive.

**Interventions:**

Given the diagnosis of HSV type 2 as the putative trigger for HLH, she was started on parenteral acyclovir for 2 weeks followed by oral valacyclovir for 2 more weeks. In the context of infection, the azathioprine was stopped while low-dose steroid and tacrolimus were continued.

**Outcomes:**

With the initiation of treatment for HSV infection, leukopenia, creatinine, and transaminases improved along with ferritin levels. At her 6-month follow-up, her blood counts and liver enzymes had normalized, and ferritin was 566 µg/L.

**Teaching points:**

Hemophagocytic lymphohistiocytosis is a rare disease in kidney transplant recipients with a high mortality rate. It can occur even in remote kidney transplant recipients so a high degree of suspicion is necessary to lead to a prompt diagnosis. Infections are common triggers for secondary HLH. Early identification and treatment of the triggering infection may improve outcomes.

## Abrege

**Justification:**

La lymphohistiocytose hémophagocytaire (HLH) est une maladie potentiellement mortelle caractérisée par une activation excessive du système immunitaire. Elle est plus fréquente chez les enfants, mais de plus en plus observée chez les adultes. La HLH primaire se manifeste en raison d’une prédisposition génétique, tandis que la HLH secondaire se développe dans le contexte d’une infection, d’un cancer ou d’une maladie auto-immune. La HLH a rarement été décrite chez les patients sous traitement immunosuppresseur à la suite d’une transplantation rénale. Nous présentons un cas de HLH déclenché par une infection virale chez une patiente avec des antécédents lointains de transplantation rénale.

**Présentation du cas:**

Une femme de 45 ans, greffée du rein en 2009 en raison d’une néphropathie à IgA, a consulté pour de la fièvre, des vomissements et des douleurs dorsales depuis une semaine. Son triple traitement immunosuppresseur consistait en des doses quotidiennes de prednisone (5 mg), d’azathioprine (100 mg) et de tacrolimus à libération prolongée (1 mg). Son taux de créatinine usuel était de 130 umol/L.

**Diagnostic:**

Les premières investigations ont révélé une anémie, de la leucopénie, un taux de créatinine sérique élevé, une transaminite et un taux de ferritine nettement élevé de 67 600 ug/L, ce qui a justifié une biopsie de la moelle osseuse pour exclure une HLH. Les résultats ont montré une plus grande proportion de cellules CD68+ (macrophages), avec plus de 5 macrophages hémophagocytaires sur 1000. Le taux de récepteurs solubles de l’IL-2 (CD25) s’établissait à 3 406 pg/ml (606 à 2 299 pg/ml), ce qui est légèrement élevé. La patiente répondait à quatre des huit critères de la HLH et présentait un score H de 223, ce qui suggérait une probabilité de 96,9 % pour un diagnostic de HLH. L’examen secondaire approfondi des possibles déclencheurs de la HLH a inclus un prélèvement sur des ulcères génitaux qui s’est avéré positif pour le virus de l’herpès simplex (HSV) de type 2. La PCR d’échantillons sanguins était également positive pour HSV de type 2.

**Interventions:**

L’infection par HSV de type 2 ayant été identifiée comme le déclencheur présumé de la HLH, la patiente a reçu un traitement parentéral d’acyclovir pendant deux semaines, suivi d’un traitement oral de valacyclovir pour deux semaines supplémentaires. Dans le contexte de l’infection, l’azathioprine a été cessée, mais le tacrolinus et les stéroïdes à faible dose ont été maintenus.

**Résultats:**

L’amorce du traitement antiviral a entraîné une amélioration de la leucopénie, du taux de créatinine et des transaminases, ainsi que des taux de ferritine. Lors du dernier suivi à 6 mois, la numérisation sanguine et les enzymes hépatiques de la patiente s’étaient normalisées et son taux de ferritine était de 566 ug/L.

**Enseignements tirés:**

La lymphohistiocytose hémophagocytaire est une maladie rare chez les greffés rénaux et elle entraîne un taux élevé de mortalité. La maladie peut survenir même chez des greffés rénaux de longue date, de sorte qu’il est nécessaire d’exercer un degré élevé de vigilance afin de poser rapidement un diagnostic. Les infections sont de fréquents déclencheurs de la HLH secondaire. Les résultats peuvent être améliorés par l’identification et le traitement précoces de l’infection ayant déclenché la maladie.

## Introduction

Hemophagocytic lymphohistiocytosis (HLH) is a life-threatening disease characterized by excessive immune activation.^1^ Even though it frequently affects infants from birth to 18 months of age, the disease can also be observed in children and adults of all ages. Hemophagocytic lymphohistiocytosis can be primary or secondary. Primary HLH either has a genetic origin (also known as familial HLH [FHL]) or is associated with genetic immunodeficiency syndromes.^2^ Secondary (or acquired HLH) occurs mostly in the context of infections, malignancies, and autoimmune diseases. Hemophagocytic lymphohistiocytosis has been rarely described in patients on immunosuppressive therapy after kidney transplant. Here, we describe a case of HLH in a patient with a remote history of kidney transplant, triggered by a viral infection.

## Presenting Concerns

Our patient is a 45-year-old female, with a history of kidney transplant in 2009 for IgA nephropathy, on triple immunosuppression consisting of daily dose of prednisone 5 mg, azathioprine 100 mg, and tacrolimus extended release1 mg. Her baseline creatinine was 130 µmol/L. She was admitted with fever, vomiting, and back pain of 1-week’s duration but denied any history of headache, respiratory symptoms, dysuria, abdominal pain, or diarrhea.

## Clinical Findings

The patient’s fever was low grade, intermittent and associated with chills. She noticed genital ulcers for the last 3 days, and they were quite painful. She had a single sexual partner and did not have any history of sexual intercourse for prior 3 months. Her physical examination was unremarkable except for multiple genital ulcers.

Initial investigations showed hemoglobin of 85 g/L, white blood cell (WBC) count of 2.4 × 10^9^/L, serum creatinine 155 µmol/L, AST of 1435 IU/L and ALT of 874 IU/L, and serum ferritin of 67 600 µg/L.

## Diagnostic Focus and Assessment

**Figure 1.**
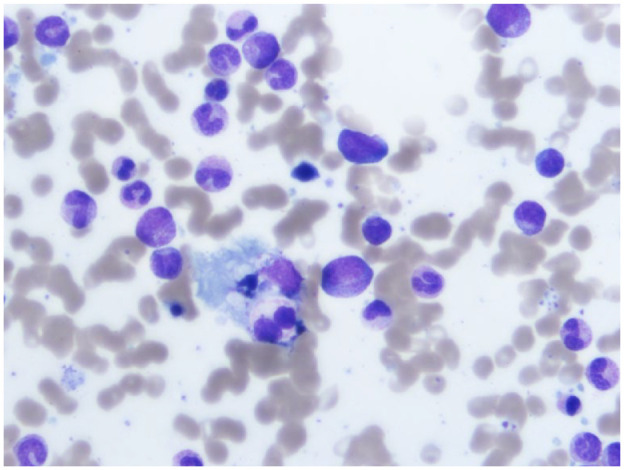
Histopathology of bone marrow section demonstrating hemophagocytosis (Wright-Giemsa stained).

The patient underwent a bone marrow biopsy due to clinical suspicion of HLH. The bone marrow showed an increased proportion of CD68+ cells (macrophages) with more than 5 in 1000 hemophagocytic macrophages (with either mature or nucleated erythrocytes, neutrophils or lymphocytes) and frequent hemosiderin laden macrophages (Figure 1). The flow cytometry showed a normal NK cell population, and her triglyceride levels and coagulation parameters (including fibrinogen levels) were within normal limits. Her soluble IL-2 receptor (CD25) level was 3406 pg/mL (606-2299 pg/mL) which was mildly elevated but did not satisfy the criteria for HLH. She fulfilled 4 of the 8 criteria for HLH, ie, fever, bicytopenia, bone marrow biopsy showing hemophagocytosis, and high ferritin (HLH-2004 [Table 1]).^3^ The H score was 223 which suggested a diagnosis of HLH with 96.9% probability.^4^ Autoimmune screening for possible triggers for HLH were negative. Malignancy workup, including computed tomography of chest, abdomen and pelvis, and serum protein electrophoresis were negative. The swab from her genital ulcer returned positive for herpes simplex virus (HSV) type 2. Polymerase chain reaction (PCR) in the blood for HSV type 2 was also positive. The other infectious screening results are provided in Table 2.

**Table 1.** Comparison of Histiocyte Society HLH-2004 and H Score Diagnostic Criteria.

HLH-2004	H score
The diagnosis HLH requires that either 1 or 2 below are fulfilled:	A higher score is associated with a higher risk of HLH, as calculated based on the following criteria and scoring system
(1) A molecular diagnosis consistent with HLH	Temperature (°C): 0 (<38.4), 33 (38.4–39.4), or 49 (>39.4)
(2) Diagnostic criteria for HLH fulfilled (5 out of the 8 criteria below)	Ferritin (ng/mL): 0 (<2000), 35 (2000-6000), or 50 (>6000)
Fever	Number of cytopenias (hemoglobin ≤9.2 gm/dL and/or leukocytes ≤ 5000/mm^3^ and/or platelets ≤110 000/mm^3^): 0 (1 lineage), 24 (2 lineages), or 34 (3 lineages)
Splenomegaly	Organomegaly: 0 (no), 23 (hepatomegaly or splenomegaly), or 38 (hepatomegaly and splenomegaly)
Cytopenias (affecting ≥2 of 3 lineages in the peripheral blood): Hemoglobin <90 g/L Platelets < 100 × 109/L Neutrophils < 1.0 × 109/L	Known underlying immunosuppression due to human immunodeficiency virus or long-term immunosuppressive therapy: 0 (no) or 18 (yes)
Hypertriglyceridemia and/or hypofibrinogenemia: Fasting triglycerides ≥ 3.0 mmol/L (ie, ≥265 mg/dL) Fibrinogen ≤ 1.5 g/L	Triglyceride (mg/dL): 0 (<132), 44 (132–353), or 64 (>353) Fibrinogen (mg/dL): 0 (>250) or 30 (≤250)
Hemophagocytosis in bone marrow or spleen or lymph nodes	Hemophagocytosis features on bone marrow aspirate: 0 (no) or 35 (yes)
Low or absent NK cell activity	Serum glutamic oxaloacetic transaminase (IU/L): 0 (<30) or 19 (≥30)
Ferritin ≥ 500 mg/L	
Soluble CD25 (ie, soluble IL-2 receptor) ≥2400 U/mL	

**Table 2.** Results of Infectious Work Up.

Investigations	Result
HSV type 2—genital ulcer swab	Positive
HSV type 2—blood PCR (12/7/23), (18/7/23)	Positive
IgM HAV	Negative
IgG HAV	Positive
Hepatitis B	Negative
Hepatitis C	Negative
Blood EBV PCR (IU/mL)—7/7/2023	17 600
Blood EBV PCR (IU/mL)—13/7/2023	95 500
Blood EBV PCR (IU/mL)—20/7/2023	54 600
CMV PCR	Negative
BKV blood PCR	Negative
Blood culture	Negative
Urine culture	Negative
AFB culture	Negative
COVID swab	Negative

Epstein-Barr Virus (EBV) viral titers at the time of presentation were 17 600 IU/mL which increased to 95 500 IU/mL at rechecking after 1 week. However, taking into account her clinical symptoms, and with the improvement in liver enzymes and ferritin levels after initiating antiviral therapy targeting HSV, EBV was not thought to be the trigger for HLH. The EBV titers continued to downtrend with serial monitoring.

## Therapeutic Focus and Assessment

The patient’s positive HSV result prompted the commencement of parenteral acyclovir for 2 weeks followed by oral valacyclovir for 2 more weeks. In the context of infection, the azathioprine was also stopped along with the initiation of antiviral treatment while low-dose steroid and tacrolimus were continued.

## Follow-up and Outcomes

**Figure 2.**
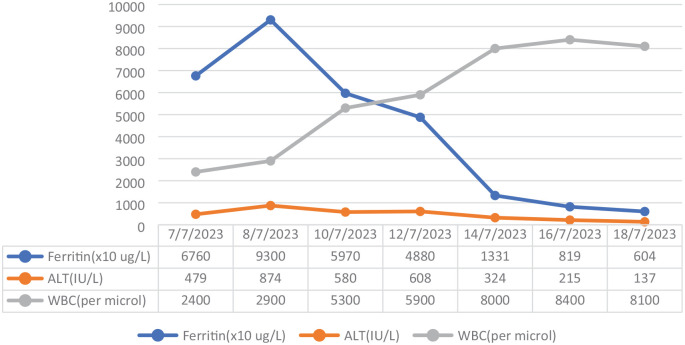
Trend of laboratory investigations during admission.

With the initiation of treatment for HSV infection, leukopenia, serum creatinine, and transaminases improved along with reduction in ferritin levels. These trends are shown in Figure 2. The patient’s azathioprine was restarted after completing her antiviral treatment. During her most recent follow-up at 6 months after the initial presentation, her blood counts and liver enzymes had normalized, ferritin was 566 µg/L, and her EBV viral load was 760 IU/mL.

## Discussion

This is, to our knowledge, the first case of HLH triggered by HSV type 2 infection in a kidney transplant recipient with recovery following treatment. Hemophagocytic lymphohistiocytosis describes a heterogeneous group of disorders characterized by sepsis-like features, typically combined with hemophagocytosis and cytopenia, impaired cellular cytotoxicity, hyperferritinemia, elevated cytokine levels, high fever, coagulation abnormalities, hepatosplenomegaly, and lymphadenopathy.^5^ Hemophagocytosis is the ingestion of cellular blood components and their precursors by macrophages, resulting from poorly controlled macrophage activity.^5^

Central to the pathogenesis is the impairment in the cytotoxic cell function (natural killer or [NK] cells and cytotoxic T cells or CTLs), leading to the inability to sufficiently eliminate antigen presenting cells (macrophages). As a consequence, there is uncontrolled expansion of macrophages and CTLs with the production of massive amounts of cytokines (cytokine storm). This results in ubiquitous inflammation and cellular organ infiltration, causing a diverse, sepsis-like syndrome. The mortality rate is highly variable, depending on the circumstances under which the HLH develops, such as underlying disorders and triggering events.^5^

Primary HLH occurs mostly in children with underlying genetic defect in genes of the cytotoxic pathways of T and NK cells.^6^ These genetic mutations cause a dysfunction of perforin-mediated cytotoxicity in 97% of familial HLH.^6^ Secondary or acquired HLH can occur at any age but generally manifests in adults in the context of infections, malignancy, or autoimmune diseases. These predisposing conditions often cause immune dysregulation and iatrogenic immunodeficiency due to immunosuppressive drug therapy (solid organ and stem cell transplantation). Sometimes the triggers are infections, such as viruses of the herpes family (Cytomegalovirus [CMV], HSV, HHV8). Notably, EBV infection is well described to be potential cause of macrophage activation. Karras et al^7^ retrospectively analyzed 17 cases of HLH after cadaveric kidney transplantation, among which viral infection was the trigger in 9 patients (CMV, EBV, HSV 6, and HSV 8). In our patient, the trigger for HLH was likely HSV type 2 as the onset of symptoms (fever) occurred in the presence of genital ulcers and her symptoms promptly responded to treatment with acyclovir along with improvement in her laboratory parameters.

Hemophagocytic lymphohistiocytosis usually presents as an acute or subacute febrile illness associated with multiple organ involvement. The diagnostic criteria for HLH was first presented by the Histiocyte Society, based on common clinical, laboratory, and histopathological findings in 1991 and later modified in 2004 with addition of 3 more criteria (Table 1). The HLH-2004 study, which included 369 patients, reported the most common symptom to be fever (95%). The common laboratory abnormalities include elevated ferritin > 500 mcg/L (94%), bicytopenia (92%), hypertriglyceridemia or hypofibrinogenemia (90%), hemophagocytosis (82%), low/absent NK cell activity (71%), and soluble CD25 elevation (97%).

The renal manifestations in HLH include acute kidney injury, subnephrotic range proteinuria, nephrotic syndrome, and chronic kidney disease. In order to characterize the kidney pathology in HLH, Sekulic et al^8^ did a retrospective review of 30 patients with HLH. Variable pathologies were identified, including acute tubular injury (ATI, 43%), lupus nephritis (LN, 23%), collapsing glomerulopathy (17%), thrombotic microangiopathy (TMA, 17%), and cortical necrosis (10%). An interesting case of interstitial hemophagocytosis in the kidney of patients with HLH was described by Sekulic et al^9^ in a patient with Sjogren’s syndrome with HLH. However, the significance of interstitial hemophagocytosis on kidney function is unclear and likely depends on the extent of interstitial infiltrates, similar to extramedullary hematopoiesis.

A scoring system has been developed to generate a diagnostic score referred to as a “H score” that estimates the probability of HLH;^4^ this incorporates points for immunosuppression, fever, organomegaly, levels of triglycerides, ferritin, alanine aminotransferase, fibrinogen, cytopenias, and presence of hemophagocytosis on the bone marrow aspirate (Table 1). An H score ≥ 250 confers a 99% probability of HLH, whereas a score of ≤ 90 confers a < 1% probability of HLH. The H score in our patient was 223, which supported the diagnosis of HLH. A study by Bilston et al^10^ attempted to compare the diagnostic accuracy of the HLH-2004 criteria and the H score as well identify optimal cutoffs stratified by underlying etiology. An external validation using a large retrospective cohort demonstrated excellent discriminatory power of both HLH-2004 criteria and H score ≥200 in predicting HLH. In the subgroup of HLH due to autoimmune etiology, the H score may add additional value to the HLH-2004 criteria for patients with borderline score via improved sensitivity. In the case series by Karres et al,^7^ death occurred in 8 of 17 kidney transplant recipients (47%) and graft nephrectomy was performed in 4 out of the 9 surviving patients. The overall mortality rate of HLH is very high, but varies among studies from 26.5% to 74.8%.^11-14^ In an analysis by Park et al,^15^ low histiocyte proportion in bone marrow and early initiation of treatment was shown to correlate with a favorable outcome. Our patient showed a complete recovery in symptoms and laboratory parameters following treatment of the triggering infection (HSV type 2). Treatment of the underlying trigger for HLH may have been initiated early enough that the cycle of immune activation was thwarted. The cessation of the antiproliferative agent (azathioprine) early at the time of presentation may also have contributed to her positive outcome. Of note, the bone marrow showed a low histiocyte proportion, suggesting a more favorable prognosis.

## Conclusion

Hemophagocytic lymphohistiocytosis is a rare disease in kidney transplant recipients with a high mortality rate. It can occur even in remote kidney transplant recipients so a high degree of suspicion is necessary to lead to prompt diagnosis. Infections are common triggers for secondary HLH and early identification and treatment of the triggering infection may improve outcomes.
